# Antibiotic prophylaxis in acute cholecystectomy revisited: results of a double-blind randomised controlled trial

**DOI:** 10.1007/s00423-020-01977-x

**Published:** 2020-08-29

**Authors:** Gona Jaafar, Gabriel Sandblom, Lars Lundell, Folke Hammarqvist

**Affiliations:** 1 Subject Trauma Reparative Medicine, CLINTEC, Karolinska University Hospital, 14186 Stockholm, Sweden; 2 Department of Clinical Science and Education, Department of Surgery, Södersjukhuset, Karolinska Institute, Stockholm, Sweden; 3 Department of Surgery, CLINTEC, Karolinska Institute, Stockholm, Sweden; 4 Department of Surgery, Odense University Hospital, J.B. Winsloews Vej 4, 5000 Odense, Denmark

**Keywords:** Acute cholecystitis, Laparoscopic cholecystectomy, Antibiotic prophylaxis, Postoperative complication, Postoperative infectious complication, Bacteriobilia

## Abstract

**Purpose:**

Evidence supporting the value of preoperative antibiotic prophylaxis (PAP) in surgery for acute cholecystitis is lacking. This study aimed to shed light on whether PAP in acute cholecystectomy for cholecystitis reduces the postoperative infectious complication (PIC) rate. Secondary outcomes were the prevalence of bacteriobilia, CRP values and leucocyte counts.

**Methods:**

The study was performed as a single-centre, double-blinded, placebo-controlled, randomised study. Patients with acute cholecystitis amenable for acute laparoscopic cholecystectomy were randomly assigned to either PAP (piperacillin/ tazobactam) or placebo, and the subsequent clinical course was followed.

**Results:**

A total of 106 patients were enrolled, 16 of whom were excluded due to protocol violation. PIC developed in 22 of the 90 patients included with no significant difference between the PAP and placebo groups (8 patients in the PAP group and 14 in the placebo arm, *p* = 0.193). The PIC rate was significantly higher in patients with a raised CRP at randomisation and on the day of surgery and in cases of conversion to an open procedure (*p* = 0.008, 0.004 and 0.017, respectively) but with no differences between the study groups.

**Conclusion:**

PAP does not affect the risk for PIC in patients with acute cholecystitis. The major risk factors determining PIC in these patients need defining, in particular, the impact of bacteriobilia.

**Trial registration:**

The study was registered at clinicaltrials.gov (NCT02619149) December 2, 2015.

## Introduction

The standard treatment for mild to moderate acute cholecystitis is early laparoscopic cholecystectomy [1–4]. Given that the overall complication rate after cholecystectomy is approximately 11% (Swedish GallRiks register) [5], the risk for postoperative infectious complications (PIC) after gallstone surgery is small but not negligible. The risk appears to be enhanced by the severity of the ongoing cholecystitis, and PIC has been shown to occur in 17% or more of patients with grades I and II acute cholecystitis [6]. The pathogenesis of PIC in connection with elective and acute operations for gallstone disease includes contamination with infected bile. Under normal conditions, bile in the gallbladder and the biliary tree is sterile [7]. Obstruction of the outflow of bile by gallstone(s) initiates an inflammatory process that eventually leads to bacterial colonisation. In acute cholecystitis, the bile becomes colonised in 35–60% of the cases [8], the most common agents being gram-negative organisms (*Escherichia coli* 31–44%, *Klebsiella* 9–20%) and *Enterococci* (3–34%) [9, 10]. The mechanisms behind PIC are complex, and the details of the dynamics of colonisation are lacking. A recent retrospective study in Sweden showed that the only significant risk factor for PIC (approximately 6%) was a positive bile culture. Gender, age, ongoing or previous cholecystitis and preoperative antibiotic prophylaxis (PAP) had no impact on PIC [11]. However, despite the fact that surgery for acute cholecystitis is one of the most frequently performed procedures by general surgeons throughout the world, the use of PAP varies between hospitals and between surgeons, as recently shown in a nationwide study from Sweden [12]. Current guidelines do not support the routine use of PAP for elective cholecystectomy for uncomplicated gallstone disease [13–15], but surprisingly adherence to international guidelines is generally low in Europe [16, 17]. Lack of evidence-based guidelines could be a significant factor behind the disparities seen worldwide. Liang et al., in a systemic review and meta-analysis of low-risk patients undergoing elective laparoscopic cholecystectomy, concluded that PAP is safe and effective in reducing surgical site infections, global infections and postoperative length of hospital stay [18]. On the other hand, the 2013 Tokyo Guidelines (TG13) recommended PAP for interventions in acute cholangitis and cholecystitis only. Implementation of TG13 has led to uniform antibiotic treatment without increasing the risk for PIC [19]. However, the effectiveness of PAP in acute cholecystitis needs to be confirmed.

The aim of this study was to determine the effect of PAP with piperacillin/ tazobactam on PIC following acute cholecystectomy for mild to moderate cholecystitis (grades I and II according to TG18).

## Material and methods

The study was undertaken as a prospective randomised double-blinded parallel group study at Karolinska University Hospital in Huddinge, with treatment allocation 1:1 between PAP and placebo.

### Eligibility criteria

Inclusion criteria were clinical and radiological signs of acute cholecystitis grades I and II suitable for acute laparoscopic cholecystectomy and age ≥ 18 years.

Exclusion criteria were ongoing septicaemia, pregnancy, bile duct obstruction, contraindication to laparoscopic surgery, treatment with antibiotic drugs within 24 h and symptom duration longer than 5 days.

Written and verbal informed consent was obtained after the decision to perform acute surgery had been taken. Inclusion and randomisation using sealed envelopes were usually performed the day before surgery, and the result was kept in a sealed envelope system by the research nurse. Blinding was performed in accordance with the recommendations of Probst et al. [20]. The intravenous drip set was covered by an opaque bag to maintain blinding, and a research nurse administered the infusion. The placebo infusion contained saline in a bottle indistinguishable from that containing the active drug. In exceptional situations, blinding was interrupted intraoperatively if the need to administer an antibiotic was considered imperative.

### Interventions and allocation

The patients were randomised to either PAP (4 g piperacillin/tazobactam) or placebo given as infusions before the procedure (79% of PAP received one dose only prior to surgery). A research nurse administrated the study infusion according to allocation. The study infusion was started immediately after inclusion and continued until the procedure was completed. As the time between inclusion and the procedure varied, infusions were given over periods varying from less than an hour to 72 h. The surgeon, ward staff, patients and researchers involved were all blinded to the allocation. Blood samples for CRP and leukocyte count were taken prior to and 2 days after the procedure by the research nurse.

### Bile sampling procedure

During the laparoscopic procedure and under sterile conditions, bile was aspirated from the fundus of the gallbladder before the start of dissection and later from the cystic duct prior to cholangiography. The bile samples were transferred to both aerobic and anaerobic sealed bottles and sent to the hospital microbiology laboratory for culture. Patients with severely inflamed cholecystitis or need for intraoperative ERCP remained in the study for intention-to-treat analyses. At Karolinska University Hospital, the common routine following laparoscopic cholecystectomy is to retrieve the specimen using a bag.

### Outcomes

The primary outcome was rate of PIC requiring antibiotic treatment or surgical intervention within 30 days postoperatively. PIC was defined as intra-abdominal abscess, sepsis, cholangitis, surgical site infection, pneumonia or urinary tract infection. Cases where antibiotic treatment was started without a clearly identified focus of infection were also classed as a PIC.

Secondary outcomes were prevalence of bacteriobilia, CRP values and leucocyte counts.

A research nurse invited all patients to a follow-up 30 days after cholecystectomy. Those who were unable to attend the follow-up were interviewed by telephone. If an event described at the telephone interview raised suspicion of a PIC, the patient’s primary care records was reviewed. The 30-day PIC rate was determined from patient’s records as well as from the patients’ own reports. Data collection was performed by the research nurses and checked by the principal investigator for correction of missing data.

### Statistical analyses and ethics

Chi-square and *t* test were used to determine differences between the two groups regarding gender, operation approach, previous gallstone symptoms, postoperative complications and comorbidity. Mann-Whitney *U* test was used for analysing non-parametric data (age, BMI, duration of symptoms, CRP, leucocyte count and body temperature). Bile culture data and inflammation markers were also analysed in relation to the occurrence of PIC. Separate per-protocol (PP) analyses were performed, i.e. excluding those who received perioperative antibiotic treatment despite placebo allocation, and those who were lost to follow-up.

### Sample size estimation:

The sample size estimation was based on a hypothesis of superiority. Assuming that PAP would reduce the risk for PIC from 25 to 10%, at least 77 patients were required to reach an 80% probability of detecting a statistically significant difference at the *p* < 0.05 level (one-sided test). This hypothesised difference was considered to reflect a clinical effect that would be clinically relevant, although a lesser reduction in the incidence of PIC may have been relevant if more serious complications were to be considered.

## Results

**Fig. 1:**
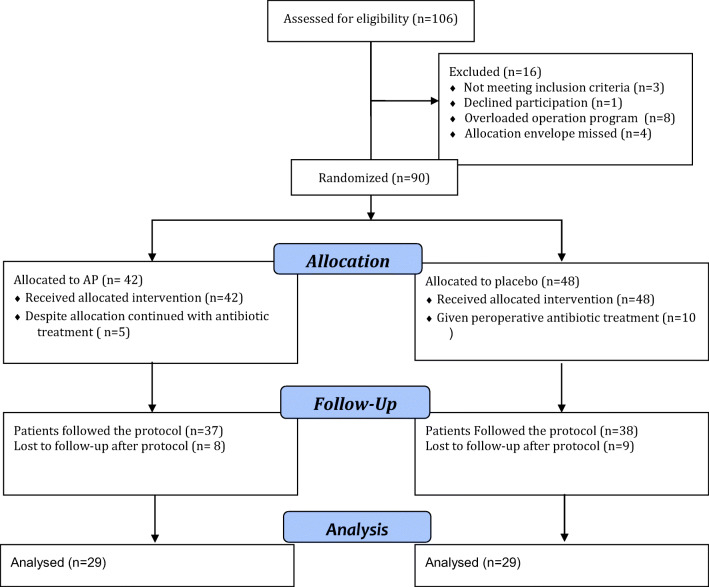
Flow chart

Altogether 106 patients were invited to participate in the study. Of these, 16 were excluded because of protocol violation (3 did not fulfil eligibility criteria, 1 withdrew consent, 8 due to postponed operation because of OR overload and 4 because the allocation envelope was missing). The remaining 90 patients were allocated to PAP or placebo (Fig. 1). The first patient was included on December 14, 2009, and the last to be included was followed until April 4, 2017. There were no differences between the groups regarding demographic and disease-specific characteristics (Table 1). Fifteen patients were given antibiotic treatment in the immediate postoperative period due to severe contamination of the abdomen, regardless of allocation. These were classified as PIC even though the decision to give antibiotic treatment was taken perioperatively whether prophylaxis had been given or not. Seventeen patients were lost to follow-up (Fig. 1). Four patients were included based on primary intent to perform laparoscopic cholecystectomy, but the surgeon responsible for the procedure decided to do an open procedure for technical reasons. These patients remained in the study.

**Table 1:** Baseline characteristics

Variables	Intention to treat analysis (*n* 90)			Per protocol analysis (*n* 58)		
	Antibiotic (%) 42 (47)	Placebo (%) 48 (53)	*p* value	Antibiotic (%) 29 (50)	Placebo (%) 29 (50)	*p* value
Men (%)	18 (43)	23 (48)	0.675	13 (45)	11 (38)	0.395
Age, years ( median, interquartile range)	48.5 (24)	49 (25)	0.768	55 (20)	45 (20)	0.194
Body mass index ( median, interquartile range)	27 (7)	28 (6)	0.874	28 (9)	27 (5)	0.428
Previous gallstone symptom (%)	13 (31)	11 (30)	0.476	10 (34)	6 (21)	0.379
No comorbidity (%)	13 (31)	21 (44)	0.277	8 (28)	14 (48)	0.175
Symptom duration (median, interquartile range)	4 (3)	4 (2)	0.653	4 (3)	4 (1)	0.178
Method of approach (%)			0.487			0.838
Laparoscopic	37 (88)	38 (79)		26 (90)	25 (86)	
Open	1 (2)	3 (6)		1 (3)	2 (7)	
Converted	4 (10)	7 (15)		2 (7)	2 (7)	
Body temperature inclusion day (median, interquartile range)	37 (21)	37 (1)	0.810	37 (2)	37 (2)	0.433
C-reactive protein inclusion day (median, interquartile range)	57 (121)	81 (129)	0.140	46 (129)	76 (79)	0.409
White blood cell count inclusion day, × 10^9^/l (median, interquartile range)	10 (5)	12 (7)	0.105	9 (6)	10.5 (8)	0.600
Temp day 2 (median, interquartile range)	37 (2)	37(0.5)	0.398	37 (2)	37 (1)	0.893
C-reactive protein day 2 (median, interquartile range)	760 (175)	80 (118)	0.650	56 (151)	70 (52)	0.844
White blood cell count inclusion day, × 10^9^/l day 2 (median, interquartile Range)	10 (7)	11 (5)	0.536	8 (8)	10 (4)	0.545
Antibiotic treatment start postop (%)	5 (12)	10 (21)	0.396	--	--	
Postop complication (%)	8 (19)	14 (29)	0.193	3 (10)	3(10)	0.665

The rate of PIC was 24% (9% in the PAP arm and 16% in the placebo arm in the intention-to-treat and 5% in each group in the PP analysis). There was no significant difference in PIC between the groups regardless of the analytical approach (*p* = 0.193, Table 2).

**Table 2:** Postoperative infectious complication

Variables	Intention to treat analysis (*N* = 90)			Per protocol analysis (*N* = 58)		
	Non-event (%) 68 (76)	Event (%) 22 (24)	*p* value	Non-event (%) 52 (90)	Event (%) 6 (10)	*p* value
Men (%)	32 (47)	9 (41)	0.633	22 (42)	2 (33)	1.000
Age (interquartile range)	47,5 (24)	58 (25)	0.081	49 (22)	59 (26)	0.301
BMI (interquartile range)	27.4 (6.5)	27.7 (6.3)	0.936	28 (7)	28 (6)	0.861
Symptom duration (interquartile range)	4 (2)	4 (3)	0.400	4 (2)	5 (4)	0.388
No comorbidity (%)	29 (43)	5 (23)	0.075 (1 s)	21(40)	1(17)	0.253 (1 s)
Operation method (%)			0.017*			0.335
Laparoscopic	61 (90)	14 (64)		46 (88)	5 (83)	
Open	2 (3)	2 (9)		2 (4)	1 (17)	
Converted	5 (7)	6 (27)		4 (8)	0 (0)	
Body temperature allocation day (median, interquartile range)	37 (2)	37 (1)	0.513	37(1)	37 (1)	0.409
C-reactive protein allocation day (median, interquartile range)	57 (121)	124 (118)	0.008*	57(94)	131 (123)	0.102
White blood cell count inclusion day, × 10^9^/l (median, interquartile range)	10 (7)	12 (5)	0.258	9 (8)	11 (3)	0.564
Body temperature day 1 (median, interquartile range)	37 (2)	37 (1)	0.560	37(2)	36 (2)	0.278
C-reactive protein day 1 (median, interquartile range)	64 (87)	206.5 (164)	0.004*	58 (63)	113 (152)	0.163
White blood cell count inclusion day, × 10^9^/l (median interquartile range )	8.5 (5)	11 (5)	0.053	8 (5)	10 (5)	0.096
*N* allocated to antibiotic prophylaxis (%)	34 (50)	8 (36)	0.193 (1 s)	26 (50)	3 (50)	0.665 (1 s)

Bile cultures were obtained in 48/90 (53%) cases (evenly distributed between the study groups), and cultures were positive in18/48 (38%) (13 in the antibiotic group and 5 in the placebo group, *p* = 0.076). Gram-negative aerobes predominated in the cultures (*N* = 11), followed by gram-positive aerobes (*N* = 10), anaerobes (*N* = 3) and fungus (*N* = 1). PIC was numerically more common in those with a positive culture, but this did not reach statistical significance (*p* = 0.054, Table 3). There was no statistically significant association between the period of PAP and a positive bile culture.

**Table 3:** Outcome of the cultures for those patients from whom bile samples were taken and rate PIC in relation to the samples

Culture (*n* = 48)			
	Positive (*n* = 18, 37.5%)	Negative (*n* = 30, 62.5%)	*p* value (1 s)
Allocation			0.076
AP	13 (72)	14 (47)	
Placebo	5 (28)	16 (53)	
Postoperative infectious complication			0.054
Non-event	12 (67)	27 (90)	
Event	6 (33)	3 (10)	

CRP levels were significantly higher in patients with PIC, on both the day of randomisation (*p* = 0.008) and the day of surgery (*p* = 0.004). PIC was also more frequently seen in cases converted to an open procedure (*p* = 0.017), whereas patient comorbidity was not associated with PIC (*p* = 0.075, Table 2).

## Discussion

In the present study, we were unable to detect any benefit of administrating PAP to reduce the risk for PIC after emergency cholecystectomy for grades I and II acute cholecystitis. Although we did not reach sufficient statistical power to detect a minor reduction in PIC rate, the absence of any significant impact speaks against the routine use of PAP. We observed a PIC rate of less than 20%: 9% in the PAP arm and 16% in the placebo arm in the ITT analysis and 5% in each group in the PP analysis. These figures compare well with the expected PIC incidences seen after emergency cholecystectomy for cholecystitis [5]. It can be argued that the preconditions applied in the sample size calculation lack clinical relevance. On the other hand, there are no definited criteria for defining clinically important differences when assessing the effectiveness of PAP. The widespread overuse of antibiotics and increasing resistance figures emphasises the importance of assessing the evidence basis of all indications for use of antibiotics. A recent randomised clinical trial, comparing single-dose antibiotic prophylaxis with single-dose AP plus an extra dose postoperatively, in patients listed for acute cholecystectomy for mild (grade I) calculus cholecystitis, demonstrated no difference in PIC rate [21]. In three other randomised trials, continuation of prophylactic antibiotic after cholecystectomy had no significant impact on PIC rate in grades I and II cholecystitis [6, 22, 23]. The results of the present study further question the use of PAP in procedures for cholecystitis.

The use of PAP in planned cholecystectomy varies between units and regions. There is still a lack of evidence supporting the use of PAP in cases of acute cholecystectomy, but even so it is obviously a generally held view that PAP reduces PIC; otherwise, the use of PAP should not be practised to such a great extent [12].

Guidelines on antibiotic use are important for all clinicians, not only to ensure proper use of antibiotics but also to minimise its overuse. Reported proportions of ESBL-producing *E. coli* range between 31.2% in two university hospitals in Germany [24] and 70.0% in a Korean university medical centre [25]. In Europe, the rate of fully susceptible *E.coli* is only 41.7% and 63.4% for Klebsiella [26]. The increasing problem of drug resistance makes evidence-based guidelines even more important. In the present study, we tested piperacillin/ tazobactam as prophylaxis. The use of piperacillin/tazobactam was motivated by the drug susceptibility pattern found in a previous Swedish study [11] and was one of the several drugs suggested in an update of the Tokyo guidelines on antimicrobial therapy for acute cholangitis and cholecystitis [10]. It could be argued, however, that its spectrum is too broad to be used for prophylaxis. Antibiotics with a spectrum more appropriate for prophylaxis, e.g*.* sulfamethoxazole/trimethoprim, could be a better alternative. Nevertheless, there are few other drugs that can be expected to be more effective as prophylaxis than piperacillin/tazobactam. Concerns other than just effectiveness must be considered when choosing an antibiotic for prophylaxis, and there is no reason to believe that the outcome of the present study would have been different if another antibiotic had been used.

As the timing of the procedure varied, the interval between study inclusions ranged from 0 to 72 h. This may have affected the impact of AP in the PAP group, although the time from the last administration of the infusion to start of surgery never exceeded 8 h since the antibiotic was administrated from the time of inclusion until surgery. There was a significant correlation between CRP at baseline and PIC rate, which may have had an impact on the outcome.

It is obvious that after procedures for acute cholecystitis, the PIC rate is significant. The mechanisms behind the development of PIC are complex including factors such as bile leakage, haemorrhage and tissue damage related to surgical trauma. Intraoperative contamination with infected bile is probably the main risk factor for PIC, which is why we specifically followed contamination and bile cultures [8–10]. We found a weak association between bacterial counts in the aspirated bile and risk for PIC which was in contrast to the risk associated with conversion to an open procedure. The rate of bile culture sampling was low, which may have affected the results of this study. The higher rate of positive cultures in the placebo group is probably due to a type I error.

We defined PIC as any infectious complication requiring drainage, surgical intervention or antibiotic treatment. This definition corresponds to grade ≥ II complications according to the Clavien-Dindo classification, but does not include surgical site infections not requiring antibiotics. This may have led to an underestimation of the PIC rate.

The double-blinded placebo-controlled design of this study ensures high internal validity. The external validity, on the other hand, is limited by the fact that it was conducted at a single centre. The blinding procedure reduced the risk for bias in the assessment of PIC. We were obliged to prolong the inclusion period due to problems in identifying and including patients that fulfilled the criteria. Another weakness of the study is that some patients were given more than one study infusion (i.e*.* antibiotic or placebo) prior to surgery, due to delay of surgery.

## Conclusion

PAP did not seem to affect the risk for PIC in patients with grades I and II acute cholecystitis. The major factors determining PIC in these patients must be better defined, in particular the role of bacteriobilia. The present study did not have sufficient statistical power to reveal minor reductions in PIC rate. PAP may also be motivated in subgroups with high risk for PIC. Further studies are needed to evaluate the effectiveness of PAP in patients undergoing surgery for acute cholecystitis.

## Data Availability

All data in the study are available on request from the corresponding author.
